# Insulin/IGF-I Signaling Pathways Enhances Tumor Cell Invasion through Bisecting GlcNAc N-glycans Modulation. An Interplay with E-Cadherin

**DOI:** 10.1371/journal.pone.0081579

**Published:** 2013-11-25

**Authors:** Julio Cesar Madureira de-Freitas-Junior, Sandra Carvalho, Ana M. Dias, Patrícia Oliveira, Joana Cabral, Raquel Seruca, Carla Oliveira, José Andrés Morgado-Díaz, Celso A. Reis, Salomé S. Pinho

**Affiliations:** 1 Division of Cellular Biology, Brazilian National Cancer Institute, Rio de Janeiro, Rio de Janeiro, Brazil; 2 Institute of Molecular Pathology and Immunology of University of Porto, Porto, Portugal; 3 Institute of Biomedical Sciences of Abel Salazar, University of Porto, Porto, Portugal; 4 Medical Faculty, University of Porto, Porto, Portugal; China Medical University, Taiwan

## Abstract

Changes in glycosylation are considered a hallmark of cancer, and one of the key targets of glycosylation modifications is E-cadherin. We and others have previously demonstrated that E-cadherin has a role in the regulation of bisecting GlcNAc *N*-glycans expression, remaining to be determined the E-cadherin-dependent signaling pathway involved in this *N*-glycans expression regulation. In this study, we analysed the impact of E-cadherin expression in the activation profile of receptor tyrosine kinases such as insulin receptor (IR) and IGF-I receptor (IGF-IR). We demonstrated that exogenous E-cadherin expression inhibits IR, IGF-IR and ERK 1/2 phosphorylation. Stimulation with insulin and IGF-I in MDA-MD-435 cancer cells overexpressing E-cadherin induces a decrease of bisecting GlcNAc *N*-glycans that was accompanied with alterations on E-cadherin cellular localization. Concomitantly, IR/IGF-IR signaling activation induced a mesenchymal-like phenotype of cancer cells together with an increased tumor cell invasion capability. Altogether, these results demonstrate an interplay between E-cadherin and IR/IGF-IR signaling as major networking players in the regulation of bisecting *N*-glycans expression, with important effects in the modulation of epithelial characteristics and tumor cell invasion. Here we provide new insights into the role that Insulin/IGF-I signaling play during cancer progression through glycosylation modifications.

## Introduction

 The Insulin Growth Factor (IGF) system in mammals comprises a dynamic network of proteins including ligands (IGF-I and IGF-II) and at least four associated receptors. The insulin receptor (IR), IGF-I receptor (IGF-IR), and insulin receptor-related receptor (IRR) belong to the tyrosine-kinase superfamily [1]. 

 Insulin/IGF-I signaling pathways play a crucial role during malignant transformation [2]. The activation of these pathways has been related with increased proliferation, survival, metastatic potential and angiogenesis [3]. Therefore, the Insulin/IGF-I signaling pathway has been considered an appealing therapeutic target in cancer [4]. In this context, it was demonstrated that tumor growth in human tumor xenograft models was significantly reduced by using antibodies that inhibit the Insulin/IGF-I signaling [5,6]. Moreover, daily treatment with OSI-906, a dual inhibitor of the IGF-I and insulin receptors, resulted in tumor growth inhibition in the NCI-H292 xenografts [7]. Furthermore, recent studies have point out the importance of the insulin/IGF-I signaling in the loss of epithelial features of carcinoma cells [8,9]. It was shown that IGF-I increases invasive potential inducing TGF-β1-mediated Epithelial to Mesenchymal Transition (EMT) in MCF-7 breast cancer cells [8]. 

E-cadherin is a cell-cell adhesion molecule with pivotal roles in the suppresion of tumor cell invasion and metastasis, being also a key molecular player in the EMT process [10]. Dysfunction of E-cadherin is considered a major event of more than 70% of human invasive carcinomas. Several mechanisms have been recently proposed to underlie E-cadherin down-regulation or inactivation in cancer, such as post-translational modifications by *N*-glycosylation [11–15]. 

It has been our long last interest to understand the role that glycans play during the carcinogenic process, particularly in the modulation and regulation of E-cadherin biological functions. In this context, we have previously demonstrated that E-cadherin functions can be specifically modulated by the presence of different oligosaccharide structures [15–17]. We have shown that during the acquisition of the malignant phenotype, E-cadherin suffered an increased modification with β1,6 GlcNAc branched *N*-glycans, catalyzed by *N*-acetylglucosaminyltransferase V (GnT-V) [18,19], that was further demonstrated to induce a destabilization of E-cadherin-mediated cell-cell adhesion (adherens junction) with consequences to tumor progression [17]. Furthermore, it was shown the existence a bidirectional cross-talk between E-cadherin expression and the *N*-acetylglucosaminyltransferase III (GnT-III) [19,20]. The modification of E-cadherin with bisecting GlcNAc *N*-glycans, catalyzed by GnT-III, was shown to enhance cell–cell adhesion with increased stability of adherens junctions, which was associated with suppression of tumor progression [17,21]. In addition, the modification of the growth receptors with bisecting GlcNAc structures precludes their membranar stabilization and consequently their signaling activation, through the inhibition of further extension and elongation of the *N*-glycans with β1,6 GlcNAc branched structures [22,23] .

 Taking into consideration the existence of a functional feedback loop between E-cadherin-mediated cell-cell adhesion and bisecting GlcNAc *N*-glycans in the suppression of cancer cell invasion, it remains to be identified which are the associated signaling pathways involved in this process. In this study, we aim to identify the E-cadherin-dependent signaling pathway involved in the regulation of *N*-glycosylation, particularly in the expression of bisecting GlcNAc *N*-glycans and their impact on the malignant phenotype of MDA-MB435 epithelial cancer cells. We herein demonstrate for the first time that on one hand E-cadherin expression induces a significant decrease in the phosphorylation levels of insulin and IGF-I receptors, which was accompanied with an increased modification of E-cadherin with bisecting GlcNAc structures, and a consequent suppression of tumor cell invasion. On the other hand, the activation of the insulin and IGF-1 signaling pathways induces a significant decrease of the bisecting GlcNAc *N*-glycans in general, and specifically on E-cadherin molecule. Concomitantly, we also observed that activation of Insulin/IGF-I signaling pathways leads to an increased tumor cell invasion. Stimulation of cancer cells with insulin and IGF-I growth factors led to a significant upregulation of the fibronectin mesenchymal marker, and an alteration of E-cadherin and β-catenin cellular localization. 

 Altogether, our results contribute to the identification of a novel molecular mechanism, involving insulin and IGF-I signaling in the modulation of bisecting GlcNAc *N*-glycans expression on E-cadherin, and their consequente impact in the modulation of the invasive phenotype. 

## Materials and Methods

### Chemicals and antibodies

Mouse monoclonal anti-E-cadherin and anti-β-catenin antibodies were obtained from BD Biosciences. Rabbit monoclonal anti-p-IR/p-IGF-IR, anti-IR, anti-IGF-IR, anti-p-Akt(Ser473), anti-Akt, anti-p-ERK 1/2, anti-ERK1/2 and anti-fibronectin were obtained from Cell Signaling Technology. Mouse monoclonal anti-α-tubulin was purchased from Sigma. Rabbit polyconal IgG anti-actin and peroxidase-conjugated anti-rabbit and anti-mouse IgG were purchased from Santa Cruz Biotechnology. *Biotinylated Phaseolus vulgaris erythroagglutinin* (E-PHA) and *biotinylated Phaseolus vulgaris leucoagglutinin* (L-PHA) lectins were purchased from Vector Laboratories. IGF-I was obtained from Immunotools and Insulin from Sigma. Alexa Fluor 488 anti-mouse was obtained from Invitrogen. 

### Cell Culture and transfection

Human MDA-MB-435 cells (which endogenously lacks E-cadherin expression at both the mRNA and protein level) were previously stably transfected with the empty vector (MDA-MB-435+mock) or with wild-type E-cadherin (MDA-MB-435+E-cad) [24]. Cells were cultured in Dulbecco’s Modified Eagle’s Medium, supplemented with 10% fetal bovine serum and 1% penicillin/streptomycin, under a humidified atmosphere containing 5% CO2. Cell lines stably transfected were maintained under antibiotic selection. MKN45 gastric carcinoma cell line stably transfected with MGAT5 or with an empty vector (mock cells) [17] were kindly provided by Prof. Taniguchi. These cells were cultured in RPMI 1640 medium containing 10% fetal bovine serum, penicillin (100 units/ml) and streptomycin (1000 μg/ml), under the selection of G418 (500 μg/ml) in 5% CO2.

### Immunoprecipitation, Western blot and lectin blot analysis

Cell cultures were washed with phosphate-buffered saline (PBS) and then lysed in a solution containing 1% Triton X-100, 1% NP40, protease inhibitor cocktail (Roche 1 tablet/50 ml buffer) and phosphatase inhibitor cocktail (Sigma, 1:100 dilution). Total protein was quantified using a BCA protein assay kit (Pierce). For immunoprecipitation, equal amounts of total protein (750 μg) from each cell lysate were precleared with 25 μl of protein G-sepharose beads (Sigma) for 1–2 h. After centrifugation, the supernatant was incubated overnight with 5 µg of mouse monoclonal antibody against E-cadherin (BD Biosciences). After that, incubation with protein G-sepharose for 2 h was performed. Next, the beads were washed three times with immunoprecipitation buffer and the immune complexes were released by boiling for 5 min at 95°C in Laemmli sampling. For Western blot, samples were subjected to 7.5% SDS–PAGE and the separated proteins were transferred to a nitrocellulose membrane. The blots were then probed with primary and pexoxidase-conjugated secondary antibodies or biotinylated lectins (Vector Laboratories). The proteins were visualized using an ECL chemiluminescence kit (GE Healthcare). Immunoreactive bands from lectin blots were then visualized using the Vector stain ABC kit (Vector Laboratories). 

### Analysis of mRNA expression by RT–PCR and real-time PCR

Total RNA from MDA-MB435+mock and MDA-MB435+E-cad cells were extracted with Tri-Reagent (Sigma) according to the manufacturer’s protocol. Yield and quality of RNA were determined spectrophotometrically. 1000 ng of total RNA were reverse transcribed using the Superscript III RNase H Reverse Transcriptase kit (Invitrogen) according to the manufacturer’s instructions. Quantitative Real-Time-PCR (qRT-PCR) was carried out in triplicates using source RNA from 3 distinct biological replicas for the target genes *CDH1* (E-cadherin, Hs01023895_m1), *Ocln* (Occludin, Hs.PT.49.14927371), *CTNNB1* (β-catenin, Hs00355045_m1), *Vim* (Vimentin, Hs.PT.47.14705389), *CDH2* (N-cadherin, Hs.PT.49.15618412), FN *1* (Fibronectin, Hs.PT.47.1565512) and for the endogenous control *GAPDH* (GAPDH, Hs.PT.51.1940505). qRT-PCR analysis of mRNA expression was performed using TaqMan Gene Expression Assays (*CDH1*, *CTNNB1*, Applied Biosystems) or PrimeTime qPCR Assays ( *Ocln*, *Vim*, *CDH2*, FN *1*, *GAPDH*, Integrated DNA Technologies). Data was analysed by the comparative 2(-ΔΔCT) method [25]. For all data comparisons, the Student's T-Test was used (two tailed, unequal variance). 

### Immunofluorescence

Cells were platted on six-well plates with coverslips. After 90% of confluence, cells were washed three times with PBS supplemented with 100 mM CaCl2 and 100 mM MgCl2 (PBS/CM), permeabilized with 0.1%Triton X-100, and blocked with BSA 3% (in PBS/CM) for one hour. For E-cadherin or β-catenin staining, cells were incubated with mouse anti-E-cadherin (BD Biosciences; 1:200 diluted in BSA 5%; one hour of incubation) or mouse anti-β-catenin (BD Biosciences; 1:100 diluted in BSA 5%; one hour of incubation) monoclonal antibody, respectively. After primary antibody incubation, the cover slips were washed in PBS and then Alexa Fluor 488 anti-mouse was used as secondary antibody (Invitrogen; 1:500 diluted in BSA 5%; one hour of incubation in the dark). Finally, the cover slips were washed in PBS and mounted on slides using Vectashield with DAPI (Vector Laboratories). Immunofluorescent images were obtained using a Zeiss Imager.Z1 AxioCam MRm (Carl Zeiss) and a TCS SP5 II (Leica) Laser Scanning Confocal microscope (Figure S4).

### Phospho-receptor tyrosine kinase array

Human Phospho-RTK Array kit for 42 RTKs was performed for both MDA-MB-435+mock and MDA-MB-435+E-cad according manufacturer’s protocol (R&D systems). The proteins were visualized using an ECL chemiluminescence kit (GE Healthcare). 

### Cell invasion assay

 Cells (5 x 10^4^) were seeded in the upper surface of transwell inserts with 8 µm pore size (Costar) coated with Matrigel for 24 h. After stimulation with insulin or IGF-I, the cells on the upper surface were removed and those from the lower surface were fixed with ethanol for 10 min, stained with DAPI and observed using an Axio Observer.Z1 microscope (Carl Zeiss). For quantification, three fields were counted for each assay.

### Densitometry and Statistical analysis

The protein levels were quantified by densitometry using LabWorks 4.6 software (Bio-Rad). The measurements were obtained from sub-exposed photographic films after quimioluminescence reaction and the values were normalized to the amount of housekeeping (actin or tubulin). Student's T-Test and one-way ANOVA were performed with GraphPad Prism 4.02 software (GraphPad Software Inc.). We considered data from three independent experiments to be statistically significant at *P*<0.05. Graphic data are presented as the mean + SEM.

## Results

### Exogenous E-cadherin expression induces an epithelial-like phenotype in MDA-MB-435 cells

**Figure 1:**
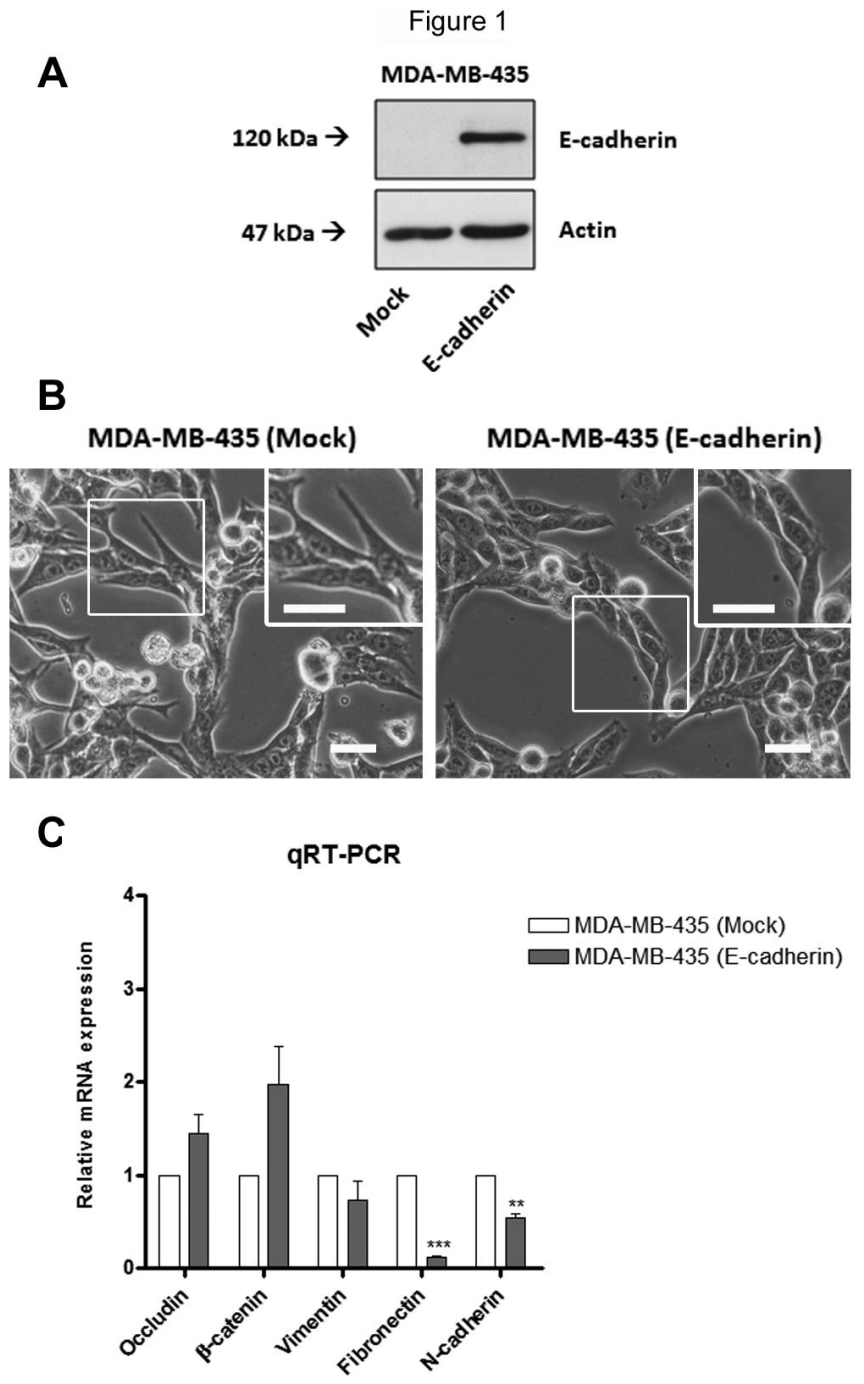
Effects of exogenous E-cadherin on the cell morfology and expression of epithelial and mesenchymal markers in MDA-MDA-435 cells. (A) Total cell lysates from MDA-MB-435+mock and MDA-MB-435+E-cad were obtained and analyzed by Western blot for E-cadherin. MDA-MB-435+mock cells do not show detectable levels of E-cadherin. Actin was used as a loading control. (B) The same amount of cells were seeded, and at the same time of culture phase contrast images show the cell morphology of MDA-MB-435+mock, which exhibit a mesenchymal-like phenotype, and MDA-MB-435+E-cad exhibit an epithelial-like phenotype. The inserts represent higher magnifications of the figure. Scale bar = 20 µm. (C) The bar graph shows the relative amount of E-cadherin, occludin, β-catenin, vimentin, fibonectin and N-cadherin mRNA levels by qRT-PCR. MDA-MB-435+E-cad cells exhibit a significant decreased expression of the mesenchymal markers fibronectin and N-cadherin. Values were normalized to the amount of mRNA in MDA-MB-435+mock. Error bars indicate the means + S.E.M. (n = 3). ** = P < 0.01, *** =P < 0.001, Student's t-test.

We used MDA-MB-435 cancer cell line, that endogenously lacks E-cadherin expression, as a model. Stable transfection with human full-length E-cadherin in MDA-MB-435 was previously performed [24]. In order to characterize the MDA-MB-435 mock and E-cadherin transfected cells, we performed a Western blot analysis using anti-Ecadherin antibody. We observed that MDA-MB-435+E-cad express E-cadherin, whereas in MDA-MB-435+mock we did not observed detectable levels of E-cadherin (Figure 1A). In addition, we have observed, by phase contrast microscopy, that expression of E-cadherin was associated with an epithelial-like phenotype whereas MDA-MB-435+mock cells exhibited a fibroblastoid-like appearance with presence of cytoplasmic protusions (Figure 1B). In order to confirm these phenotypical changes induced by E-cadherin transfection, we performed qRT-PCR to determine the expression levels of epithelial (occludin and β-catenin) and mesenchymal (vimentin, fibronectin and N-cadherin) markers at the mRNA level [26]. We observed that, the MDA-MB-435+E-cad cells exhibited a significant decreased expression of mesenchymal markers, such as fibronectin and N-cadherin, comparing with MDA-MB-435+mock. (Figure 1C). These results showed that, as expected, induced over-expression of E-cadherin inhibits the spindle shape morphology and induces an epithelial-like phenotype in MDA-MB-435 cells.

### Exogenous E-cadherin expression inhibits insulin/IGF-I signaling pathways in MDA-MB-435 cells

**Figure 2:**
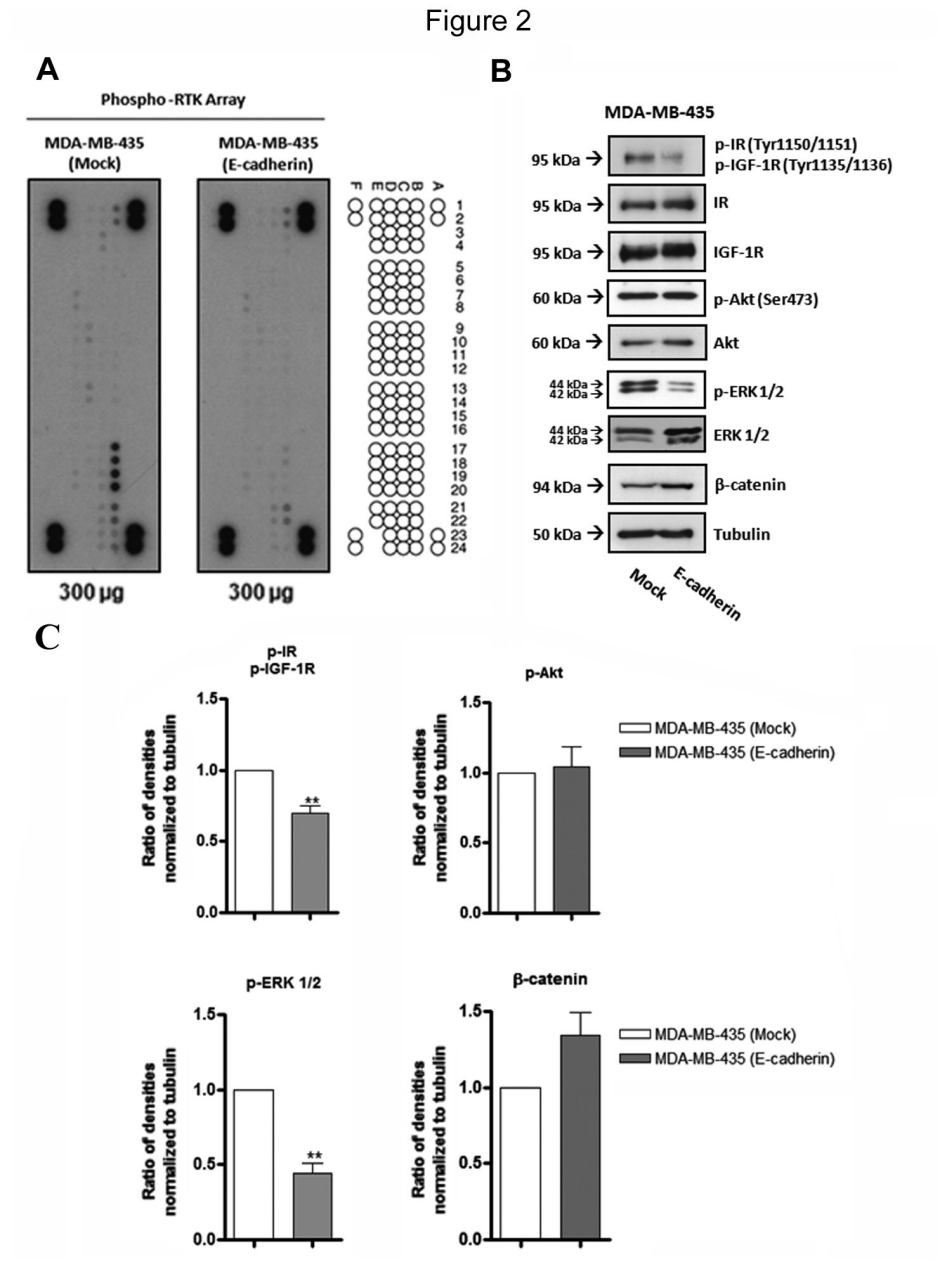
Effects of exogenous E-cadherin expression on the phosphoproteome profile of tyrosine kinase receptors and downstream proteins. (A) Total cell lysates from MDA-MB-435+mock and MDA-MB-435+E-cad were obtained and analyzed by Phospho-RTK array using 300 µg of proteins. The phosphor-RTK coordinates are shown on the right side of figure illustrating the localization of the spots containing immobilized antibodies on the nitrocellulose membrane. B17 and B18 represent the IR spots, whereas B19 and B20 represent IGF-IR spots, both receptors show decreased phosphorylation levels upon E-cadherin expression. (B) Total cell lysates from MDA-MB-435+mock and MDA-MB-435+E-cad were obtained and analyzed by Western blot for phospho-IR(Tyr1150-51)/phospho-IGF-IR(Tyr1135-36), IR, IGFR, Akt, phospho-Akt (Ser 473), ERK 1/2, phospho-ERK 1/2 and β-catenin. MDA-MB-435+E-cad cells show significant decreased levels of phospho-IR/phospho-IGF-IR and phospho-ERK1/2, comparing with mock cells. Tubulin was used as a loading control. The bar graphs show the relative amount of proteins levels normalized to tubulin. Error bars indicate the means + S.E.M. (n = 3). ** = P < 0.01, Student's t-test.

Activity of receptors tyrosine kinase (RTK) has been implicated in Epithelial-to-mesenchymal transition (EMT) program, which is turn is closely associated with tumor cell invasion and metastases [27]. We evaluated the impact of E-cadherin expression in the phosphorylation profile of RTK, by performing a phosphoproteome-array. Our results showed a marked decrease in the phosphorylation levels of insulin receptor and IGF-I receptor upon E-cadherin expression (Figure 2A and Figure S1). These results were further validated by Western blot analysis, showing a significant (*P*<0.01) decrease of phospho-IR(Tyr1150-51)/phospho-IGF-IR(Tyr1135-36) (Figure 2B and C). IR and IGF-IR can activate different downstream signaling pathways such as, Ras/Raf/MEK/ERK; PI3K/Akt and β-catenin [5,9]. In order to identify which signaling pathway is being modulated by exogenous E-cadherin expression, we analysed the protein expression levels of phospho-ERK1/2, total ERK1/2, phospho-Akt (Ser473), total Akt and β-catenin. The results demonstrated that E-cadherin expression led to a significant (*P*<0.01) decrease of ERK1/2 phosphorylation, and no significant changes of phospho-Akt (Ser473) were observed. No changes were observed for the expression of total Akt and ERK. In addition, we observed a slight increase, of the β-catenin protein expression levels upon induced E-cadherin over-expression (Figure 2B and C). These results suggest a relationship between E-cadherin expression and the modulation of IR/IGF-IR phosphorylation levels. In addition, the impact on the phosphorylation levels of ERK1/2 after E-cadherin expression further support that, in this cancer cell model, the IR/IGF-IR-mediated downstream pathway that is preferentially activated is the Ras/Raf/MEK/ERK. 

### Stimulation with insulin and IGF-I growth factors restore the phosphorylation of IR, IGF-IR and ERK 1/2, leading to cytoplasmic expression of E-cadherin and β-catenin

**Figure 3:**
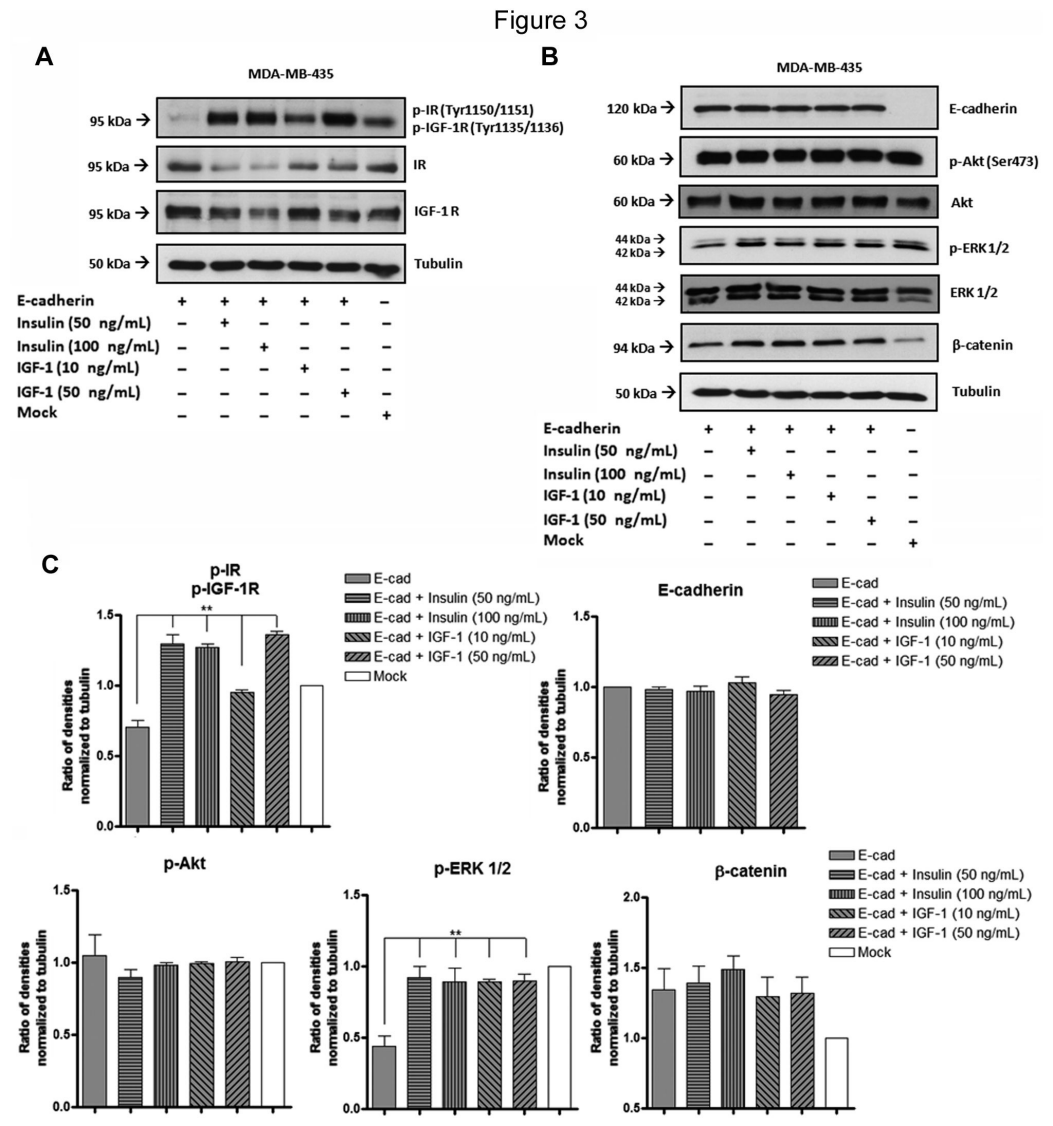
Effects of stimulation with insulin and IGF-I on the phosphorylation of tyrosine kinase receptors and downstream proteins. (A,B) Total cell lysates from MDA-MB-435+mock, MDA-MB-435+E-cad and MDA-MB-435+E-cad stimulated (24h) with insulin or IGF-1 were obtained and analyzed by Western blot for phospho-IR(Tyr1150-51)/phospho-IGF-IR(Tyr1135-36), IR, IGFR, Akt, phospho-Akt (Ser 473), ERK 1/2, phospho-ERK 1/2, β-catenin and E-cadherin. Increased phosphorylation levels of IR, IGF-IR, and ERK 1/2 were observed after stimulation with insulin or IGF-I. Tubulin was used as a loading control. No changes were observed for the total expression levels of Akt and ERK1/2. (C) The bar graphs show the relative amount of proteins levels normalized to tubulin. Error bars indicate the means + S.E.M. (n = 3). ** = P < 0.01, ANOVA t-test.

**Figure 4:**
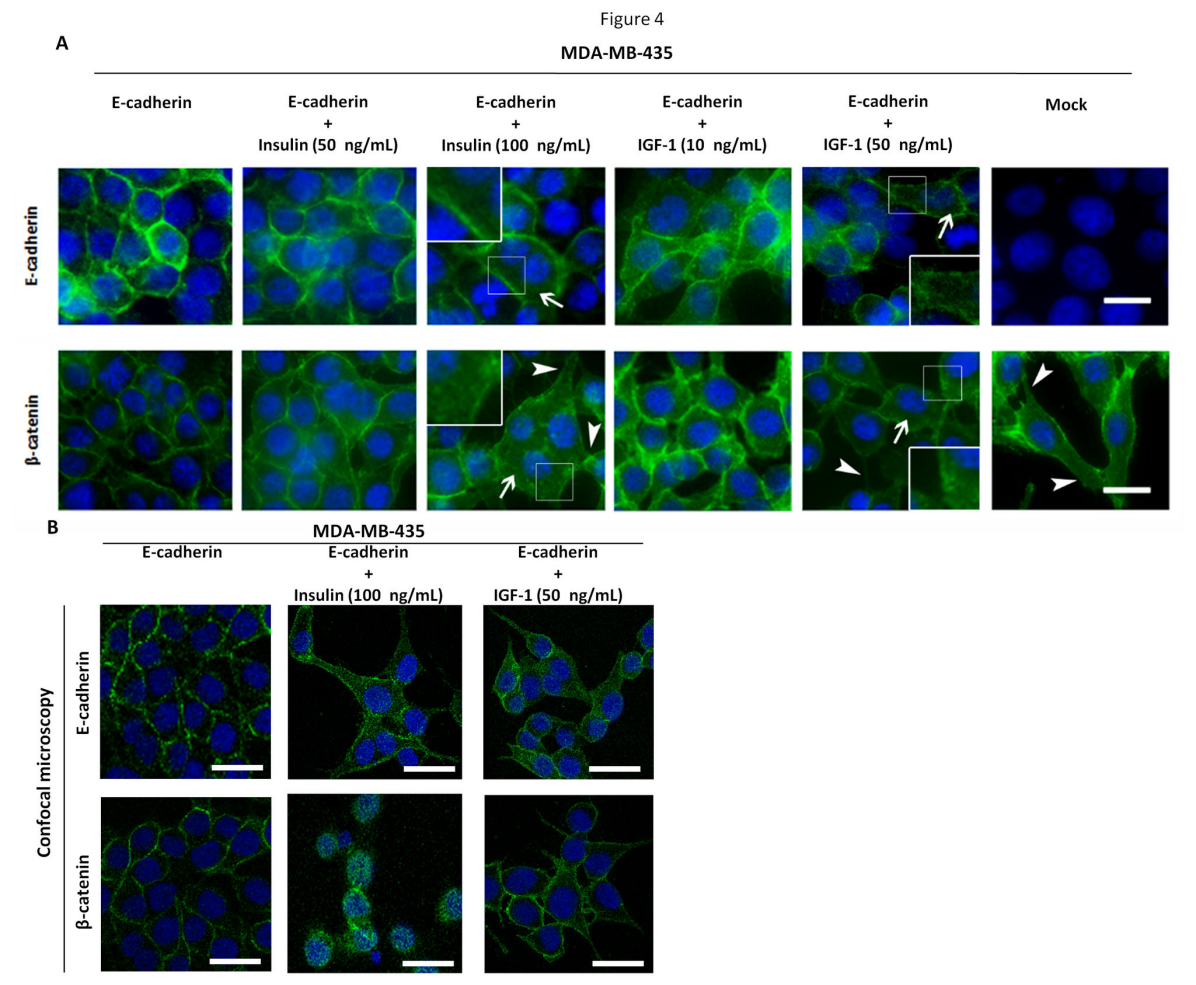
Subcellular localization of E-cadherin and β-catenin after stimulation with insulin and IGF-I. (A) Cell monolayers from MDA-MB-435+mock, MDA-MB-435+E-cad and MDA-MB-435+E-cad stimulated (24h) with insulin or IGF-1 were fixed and stained for E-cadherin, β-catenin and nucleus (DAPI). Diffuse cytoplasmic expression levels of β-catenin and E-cadherin are observed (arrows) after stimulation with insulin or IGF-I, together with observation of cytoplasmic protusions compatible with a fibroblastoid-like appearance (arrowheads). The representative images were obtained by fluorescence microscopy. White arrows and magnified images indicate cytoplasmic staining. Bar = 10 µm. (B) Confocal microscopy showing the cytoplasmic staining of E-cadherin and β-catenin after stimulation with insulin and IGF-I. Bar = 10 µm.

MDA-MB-435+E-cad were treated with growing concentrations of insulin and IGF-I growth factors. We first evaluated by Western blot whether these growth factors were able to restore phosphorylation levels of IR/IGF-IR and their downstream signaling pathway. The results showed that both, insulin and IGF-I, significantly (*P*<0.01) increased the phosphorylation levels of IR/IGF-IR (Figure 3A and 3C) and the phosphorylation levels of ERK 1/2 (*P*<0.01) in MDA-MB-435+E-cad cells (Figure 3B and 3C). Stimulation with insulin and IGF-I were not able to modulate the levels of phosphorylated Akt in our cell line model. Moreover, no significant changes were observed in E-cadherin and β-catenin proteins levels (Figure 3B and 3C). After stimulation with insulin or IGF-I, we have observed an impact in the cellular localization of E-cadherin and β-catenin. The results showed that upon insulin/IGF-I treatment there was a trend to an increased diffuse cytoplasmic expression of E-cadherin and β-catenin, as observed by immunofluorescence, together with some alterations in cellular morphology (appearance of cytoplasmic protusions, compatible with mesenchymal-like features) (Figure 4). 

### Activation of Insulin and IGF-I signaling pathways induces a decrease in the bisecting GlcNAc N-glycans expression in general and specifically on E-cadherin

**Figure 5:**
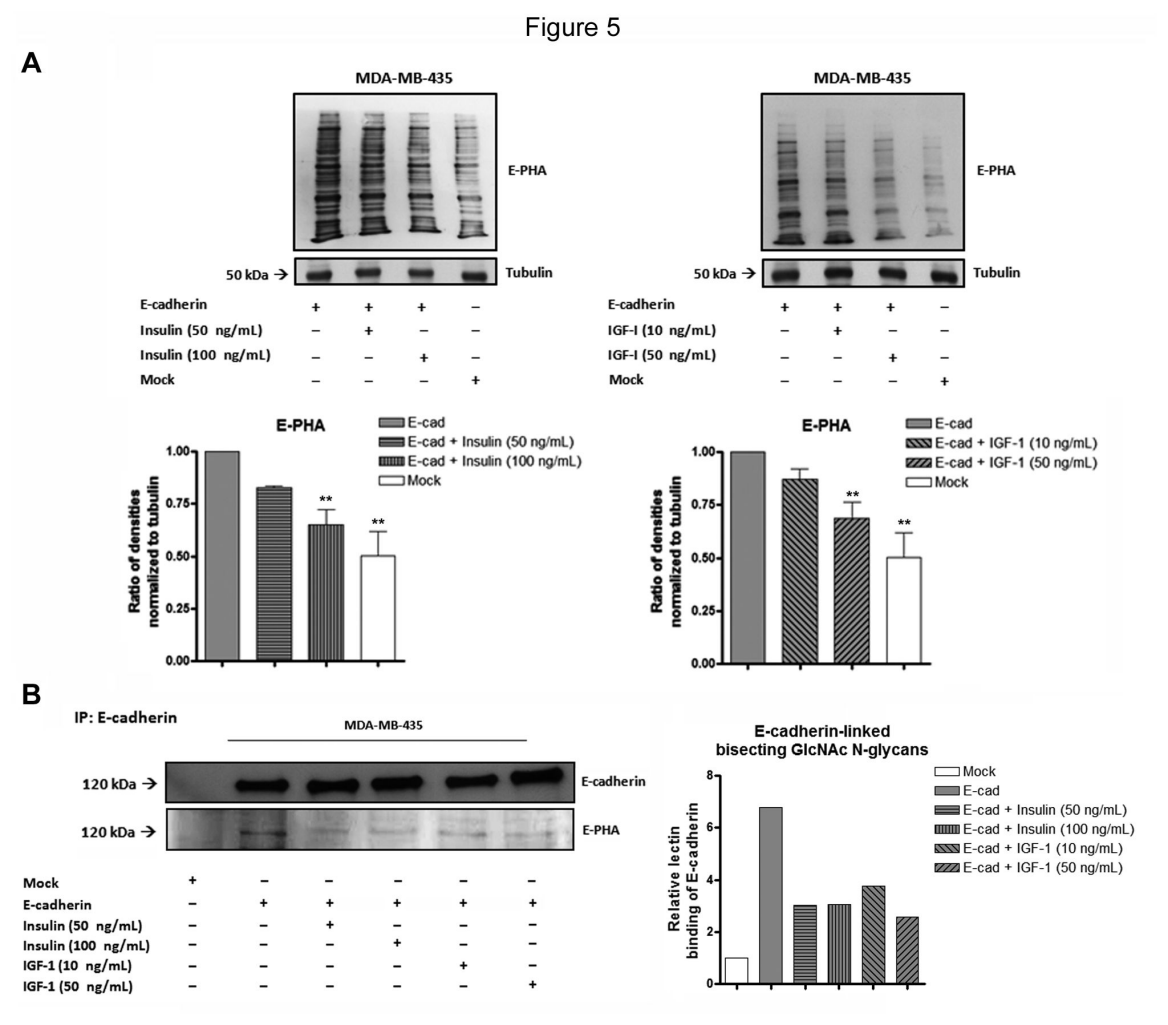
Effects of insulin and IGF-I stimulation on the expression levels of bisecting GlcNAc *N*-glycans, in general and specifically on E-cadherin. (A) Total cell lysates from MDA-MB-435+mock, MDA-MB-435+E-cad and MDA-MB-435+E-cad stimulated (24h) with insulin or IGF-1 were obtained and analyzed by Lectin blot for E-PHA. The bar graphs show the relative amount of bisecting GlcNAc *N*-glycans levels in the whole protein lysate. MDA-MB-435+E-cad cells stimulated with insulin (100 ng/mL) and IGF-I (50 ng/mL) showed a significant decrease of the overall levels of bisecting GlcNAc N-glycans. The values were normalized to tubulin. Error bars indicate the means + S.E.M. (n = 3). ** = P < 0.01 ANOVA test. (B) Total cell lysates from MDA-MB-435+mock, MDA-MB-435+E-cad and MDA-MB-435+E-cad stimulated (24h) with insulin or IGF-1 were obtained and immunoprecipitated using E-cadherin antibody. The immunoprecipitates were analyzed by Western blot for E-cadherin and Lectin blot for E-PHA. The bar graphs show the relative amount of E-cadherin-linked bisecting GlcNAc *N*-glycans levels. Activation of insulin and IGF-I signaling pathway led to a decreased modification of E-cadherin with bisecting GlcNAc N-glycan structures.

Previous studies have demonstrated that E-cadherin induces an increased expression of bisecting GlcNAc *N*-glycans [19], which in turn is associated with an increased stability of E-cadherin-mediated cell-cell adhesion [17]. However, it remained to be determined the E-cadherin-dependent signaling pathway involved in this glycosylation modulation. Taking into consideration the inhibition of insulin/IGF-I signaling pathways induced by E-cadherin expression, we went further and assessed the impact of Insulin and IGF-I signaling pathways in the modulation of bisecting GlcNAc *N*-glycans expression, in MDA-MB-435 cancer cells. We first evaluated, by lectin blot analysis, the impact of insulin and IGF-I stimulation in the total expression levels of bisecting GlcNAc *N*-glycans [21,28]. We observed that both insulin (100 ng/mL) and IGF-I (50 ng/mL) stimulation of MDA-MB-435+E-cad cells induced a significant decrease (*P*<0.01) of the overall levels of bisecting GlcNAc *N*-glycans (Figure 5A). The levels of expresion of bisecting GlcNAc *N-*glycans in MDA-MB-435+Ecad cells stimulated with higher concentrations of insulin and IGF are similar to those expressed in mock cells. Furthermore, we evaluated the effect of this bisecting GlcNAc *N-*glycans modulation specifically on E-cadherin molecule. This was performed by immunoprecipitation of E-cadherin followed by lectin blot analysis using E-PHA lectin, that recognizes the bisecting GlcNAc structures. The results showed that activation of insulin and IGF-I signaling pathway led to a decreased modification of E-cadherin with bisecting GlcNAc structures (Figure 5B). 

### Stimulation of MDA-MB-435+E-cad cells with Insulin and IGF-I up-regulates mesenchymal markers and enhances tumor cell invasion

**Figure 6:**
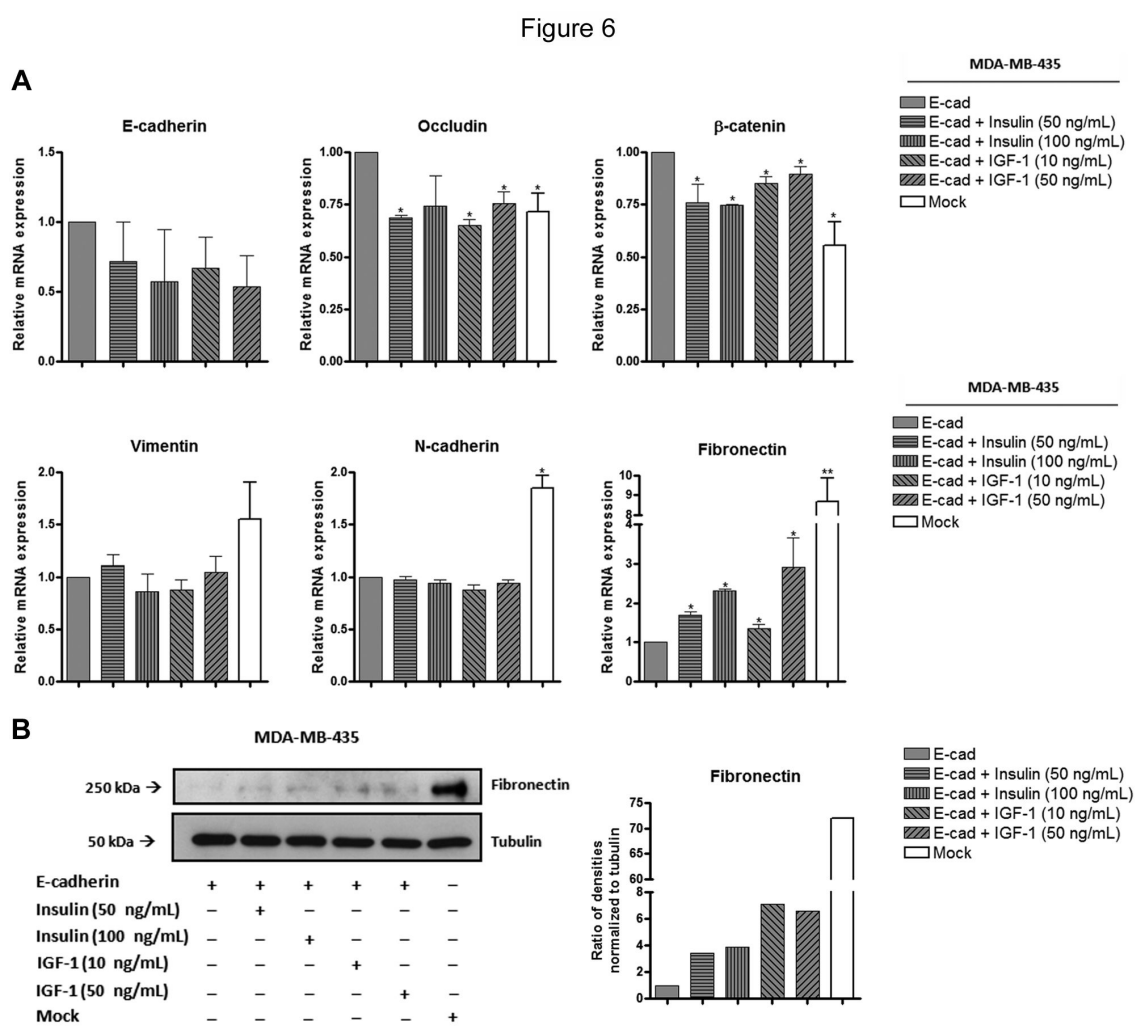
Effects of insulin and IGF-I stimulation on the mRNA expression levels of epithelial and mesenchymal markers. (A) The bar graphs show the relative amount of E-cadherin, occludin, β-catenin, vimentin, fibronectin and N-cadherin mRNA levels by qRT-PCR. Significant down-regulation of the mRNA levels of epithelial markers (occludin and β-catenin) and an up-regulation of the mesenchymal marker fibronectin were observed after insulin and IGF-I stimulation. Values were normalized to the amount of mRNA in MDA-MB-435+E-cad. Error bars indicate the means + S.E.M. (n = 3). * = P < 0.05, ** = P < 0.01, ANOVA test. **Effects of stimulation with insulin and IGF-I on the fibronectin protein levels**. (B) Total cell lysates from MDA-MB-435+E-cad,MDA-MB-435+E-cad stimulated (24h) with insulin or IGF-1, and MDA-MB-435+mock were obtained and analyzed by Western blot for fibronectin. Increased fibronectin expression levels are observed after stimulation with insulin or IGF-I. The bar graphs show the relative amount of fibronectin levels normalized to tubulin.

**Figure 7:**
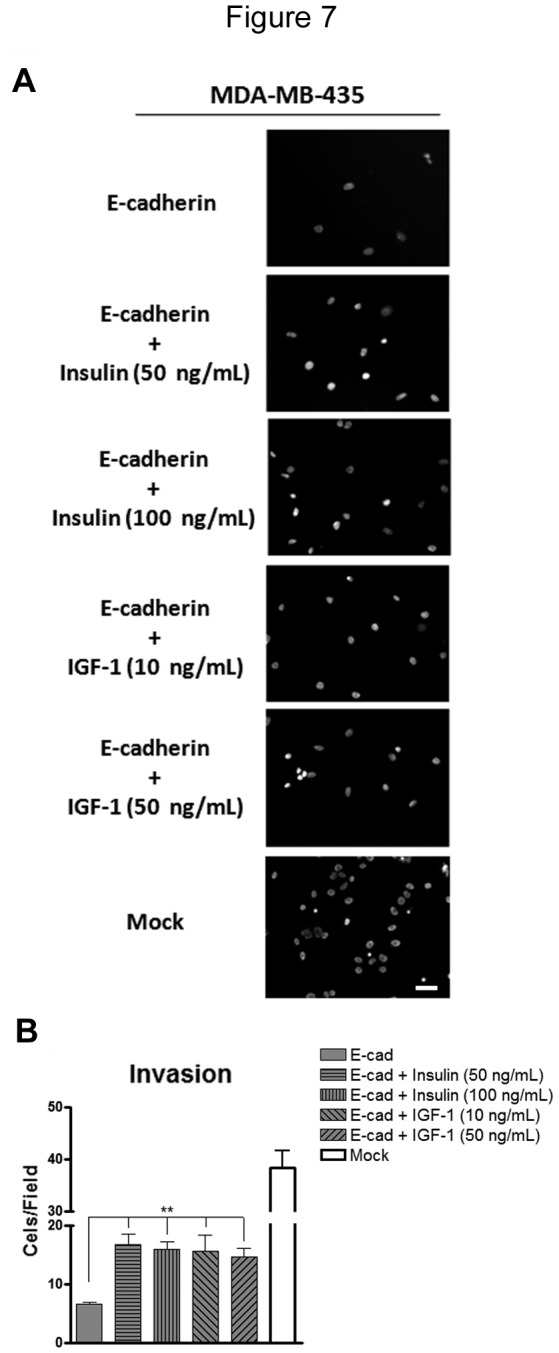
Effects of stimulation with insulin and IGF-I on cell invasion. Representative images of cell invasion through Matrigel using 8 mm pore of a polycarbonate membrane. Nuclei were stained with DAPI. We observed a significant increase of tumor cell invasion of MDA-MB-435+Ecad after stimulation with insulin or IGF-I. The bar graph shows the amount of cells/field. Error bars indicate the means + S.E.M. (n = 3). ** = P < 0.01 ANOVA test.

Since we observed a reduction of E-cadherin modification with bisecting GlcNAc *N-*glycans upon insulin and IGF-I treatment, which was accompanied with alterations in E-cadherin cellular localization and some alterations in cell morphology, we next assessed the impact of insulin and IGF-I signaling in cell differentiation and cellular function of MDA-MB-435 cells expressing E-cadherin. We evaluated the effect of insulin and IGF-I stimulation in the expression profile of EMT markers as well as in tumor cell invasion. Our results showed that both insulin and IGF-I stimulation induced a significant down-regulation of the mRNA levels of some epithelial markers (occludin and β-catenin) and an up-regulation of the mesenchymal marker fibronectin as evaluated at the mRNA levels (Figure 6A). Corroborating this result, stimulation with insulin and IGF-I also increased the fibronectin expression at the protein level, which has been closely associated with tumor cell invasion [27] (Figure 6B). E-cadherin, vimentin and N-cadherin did not undergo significant changes at the mRNA levels (Figure 6A). Interestingly, our results further showed that activation of insulin and IGF-I signaling, through growth factor stimulation, significantly enhanced (*P*<0.01) tumor cell invasion of MDA-MB-435+Ecad (Figure 7). Taken together, our results demonstrated that insulin and IGF-I signaling could in fact promote an invasive phenotype by changing the bisecting GlcNAc *N-*glycosylation profile of E-cadherin and consequently the expression pattern of epithelial/mesenchymal markers of cancer cells. 

## Discussion

 Insulin/IGF-I signaling pathways have been widely implicated in the processs of tumor development and progression of different types of cancer being considered an important target with promising therapeutic aplications in cancer. 

 Aberrant glycosylation is considered as a key event in the process of tumor cell invasion and metastasis [29]. One of the most widely occurring glycosylation changes inducing malignancy is enhanced β1,6GlcNAc branching of *N*-linked structure, catalyzed by *N*-acetylglucosaminyltransferase V (GnT-V), which is counteracted by the synthesis of bisecting GlcNAc *N-*glycans catalyzed by *N*-acetylglucosaminyltransferase III (GnT-III) [28]. Increased bisecting GlcNAc *N-*glycans inhibits β1,6GlcNAc branching, leading to suppression of metastasis [20]. In this process, one of the target molecules is E-cadherin, in which the bisecting GlcNAc *N*-glycans is associated with enhanced cell-cell adhesion and increased stability of intercellular adherens-junctions, consequently contributing to suppression of tumor progression [15,17,21,30,31]. Previously, we and others have shown that the E-cadherin glycoprotein has a role in the regulation of bisecting GlcNAc *N*-glycans expression [19]. However, it remained to identify which signaling pathways (E-cadherin-dependent) were involved in this regulation of glycosylation, and associated with tumor supression. In the present study we aimed to assess the underlying signaling pathway (E-cadherin-dependent) involved in the regulation of the bisecting GlcNAc *N*-glycans in the process of tumor progression of epithelial cancer cells. 

 We have evaluated the impact of E-cadherin *de novo* expression in the activity of different receptors tyrosine kinase using an epithelial cancer cell model. Our results demonstrated that MDA-MB-435 cancer cells, lacking endogenous E-cadherin expression, exhibited a significant increased phosphorylation of IR/IGF-IR RTK showing also decreased levels of bisecting GlcNAc *N*-glycan structures. Upon exogenous overexpression of E-cadherin, there was a remarkable inhibition of the IR/IGF-IR phosphorylation suggesting the existence of an inhibitory effect of E-cadherin in the activity of Insulin and IGF-I signaling pathways. In fact, and in accordance with our observation, in hormone refractory PC-3 prostate cancer cells, IGF-I induced a decrease of E-cadherin expression levels [9]. However, in hepatocellular carcinoma cells the scenario observed seems to be the contrary, since the epithelial phenotype was strongly associated with expression of IGF-2 and IR as well as activation of IGF-1R and IR [32]. These conflicting observations suggest that the phosphorylation pattern of IR/IGF-IR appears to be tissue/cell-specific.

 Furthermore, our results showed that ERK 1/2 protein also undergoes a significant decrease in the phosphorylation levels upon E-cadherin expression, while no changes were observed in phospho-Akt (Ser473). These observations suggest that in MDA-MD-435 cancer cells, E-cadherin expression induces a downregulation of Ras/Raf/MEK/ERK downstream pathway through the significant decreased activity of the IR/IGF-IR signaling. 

The activation of the IR/IGF-IR signaling and the downstream pathway Ras/Raf/MEK/ERK by insulin and IGF-I treatment led to a slight increase in the cytoplasmic expression of E-cadherin and β-catenin, that was accompanied with some alterations of cell morphology with observation of cytoplasmic extrusion compatible with an acquisition of a fibroblastoid-like appearance. However, this variation in cellular localization after stimulation with insulin or IGF-I appears to be not due to changes at E-cadherin and β-catenin protein levels (Figure 3). Curiously, after stimulation with Insulin or IGF-I, the mRNA levels of β-catenin underwent a significant reduction. This relationship between IR/IGF-IR signaling and β-catenin localization could be associated with a possible involvement of the activation of the WNT signaling pathway, as previoulsy described [33]. However, the mechanism underlying such modulation in the β-catenin expression need to be further investigated. Having identified IR/IGF-IR as one of the signaling pathways which activation is modulated by E-cadherin expression, we further evaluated the impact of this pathway in the regulation of glycosylation particularly the bisecting GlcNAc *N*-glycans, whose expression was described to be, in turn, regulated in an E-cadherin-dependent manner [19]. We have showed that Insulin and IGF-I stimulation induced a significant decrease of the overall levels of bisecting GlcNAc *N*-glycans expression. Furthermore, we demonstrated that this decrease through IR/IGF-IR signaling specifically targets E-cadherin molecule inducing a decreased modification of these bisecting GlcNAc *N*-glycans attached to E-cadherin glycoprotein. To the best of our knowledge, this is the first study showing such a relationship between IR/IGF-IR signaling and the modulation of bisecting GlcNAc *N*-glycans expression in general and specifically on E-cadherin. Interestingly, we further observed that IR/IGF-IR activation induced a significant increased tumor invasion capability of cancer cells. In addition, the signaling activation of IR/IGF-IR concomitantly induced modulation of expression of epithelial *versus* mesenchymal markers. We showed that IR/IFG-IR signaling activation induced an increased expression of the mesenchymal marker fibronectin (both at protein and mRNA levels), together with a decreased expression of the epithelial marker occludin. These results are in agreement with some reports describing that cell motility and proliferation have been associated with activation of MEK/ERK by Insulin/IGF-I ligands [34]. In addition, our observations are in accordance with reports showing that the autocrine production of insulin-like growth factor-I (IGF-I) reduces occludin levels and alters paracellular transport in mammary epithelial cells in vitro [35]. 

**Figure 8:**
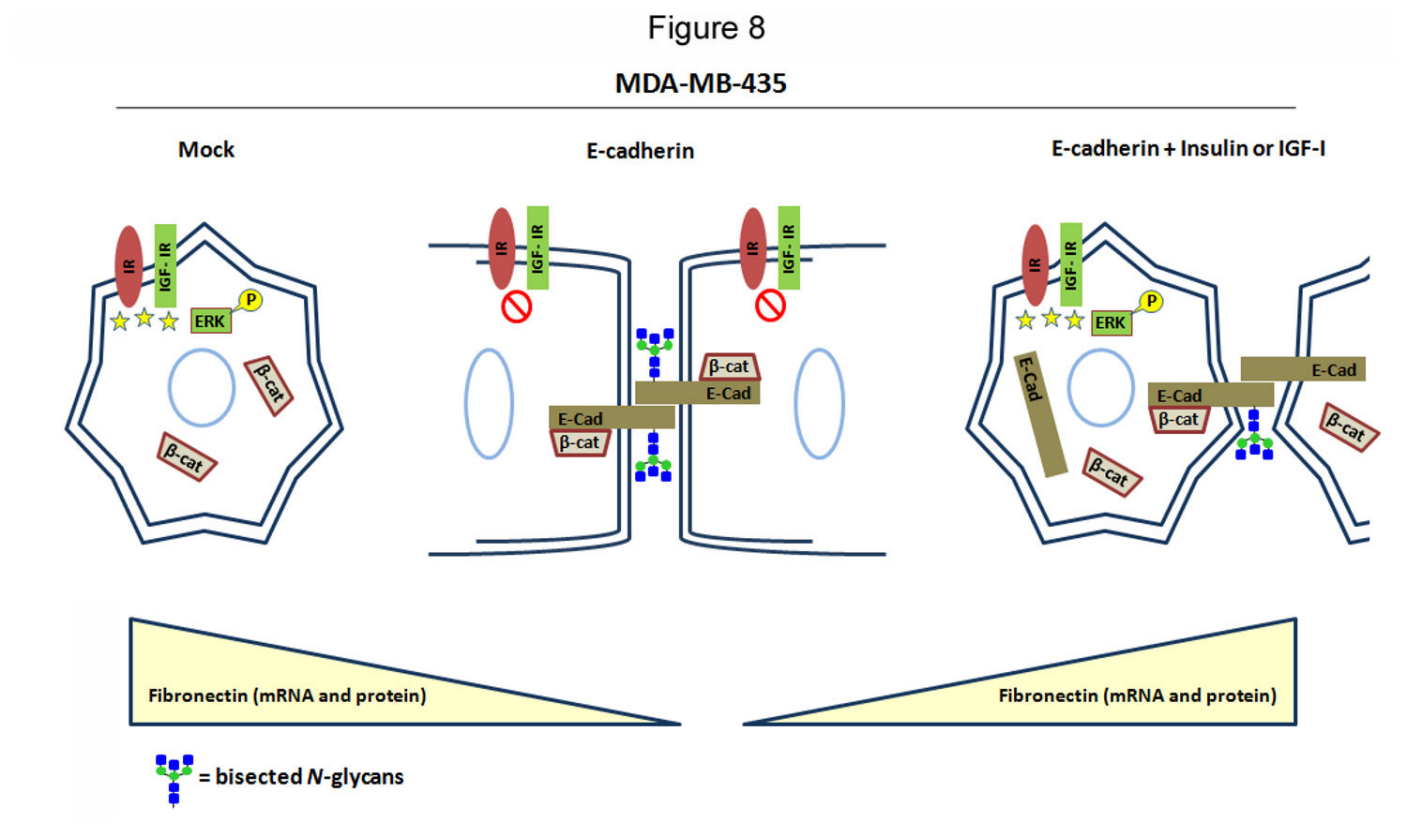
Proposing model for the interplay between E-cadherin, IR/IGF-IR, and bisecting GlcNAc *N*-glycans on the stabilization of both cell-cell adhesion and epithelial-like phenotype. The figure summarizes our findings and shows that exogenous E-cadherin expression leads to an inhibition of IR/IGF-IR signaling, concomitantly with increased levels of bisecting GlcNAc *N*-glycans expression which were previously shown to stabilizes adherens-junctions, ensuring an epithelial-like phenotype [18,35]. Stimulation with insulin or IGF-I activates IR/IGF-IR signaling and downstream protein ERK 1/2, promoting a decreased expression of bisecting GlcNAc structures in general and specifically on E-cadherin which was previously shown to destabilize cell-cell adhesion [18], leading to an invasive phenotype. Concomitantly, it was observed an increased expression of the mesenchymal marker fibronectin and cytoplasmic β-catenin and E-cadherin.

Although we cannot exclude that IR/IGF-IR signaling pathways may affect other important factors, the combination of previous reports from our and other groups [17,19,20] with the present results support a close interplay between E-cadherin, its glycosylation with bisecting GlcNAc N-glycans and IR/IGF-IR signaling in the process of tumour cell invasion. Corroborating this hypothesis, the stimulation of mock-transfected cells with insulin and IGF-I (Figures S2, S3 and S4) did not affect the fibronectin mRNA transcription levels nor the invasive phenotype (Figure S3). In addition, no changes were observed on the β-catenin cellular localization after stimulation of mock cells with insulin or IGF-I (Figure S4). Moreover, in MKN45 gastric cancer cells transfected with MGAT5 (a cellular model known to induce changes in E-cadherin glycosylation and consequently in tumour cell behaviour[17]), we have demonstrated that the overexpression of GnT-V leads to an increased expression of the Insulin Receptor (Figure S3 E), which further supports the relationship between post-translational modifications of E-cadherin and IR/IGF-IR signaling in the process of tumour cell invasion. Our observations are the first to clearly demonstrate that bisecting GlcNAc *N*-glycans expression appears to be regulated by the interplay of E-cadherin and IR/IGF-IR signaling, which are known to be crucial in the process of epithelial-mesenchymal transitions and consequetly in the process of tumor cell invasion and metastases. We here propose that E-cadherin induces the expression of bisecting GlcNAc N-glycans in the absence (switch-off) of IR/IGF-IR signaling, which in turn will contribute to the stabilization of E-cadherin at the cell membrane, ensuring an epithelial-like phenotype, ultimatly promoting tumor suppression [17,36]. The signaling activation of IR/IGF-IR pathway, through the activation of Ras/Raf/MEK/ERK, induces a significant decreased expression of bisecting GlcNAc *N*-glycans in general and specifically on E-cadherin, which in turn leads to a destabilization of E-cadherin at the cell mebrane together with increased expression of mesenchymal markers. This alteration of E-cadherin glycosylation through IR/IGF-IR signaling activation favors an increased tumor cell invasion capability (Figure 8, proposed model). 

In conclusion, our findings strongly support that Insulin/IGF-I signaling is an appealing target with implication in the modulation of glycosylation of key molecules involved in tumor invasion, having therefore promising therapeutic aplications in epithelial cancers.

## Supporting Information

Figure S1 **Effects of exogenous E-cadherin expression on the phosphoproteome profile.** Total cell lysates from MDA-MB-435+mock and MDA-MB-435+E-cad were obtained and analyzed by Phospho-RTK array using 300 µg of proteins. The phosphor-RTK coordinates are shown on the top of figure illustrating the localization of the spots containing immobilized antibodies on the nitrocellulose membrane. The bar graphs show the relative densities of black dots. The most pronounced changes are observed in IR (coordinates B17 and B18) and IGF-IR (coordinates B19 and B20).
(TIF)

Figure S2 **Effects of stimulation of Mock-transfected cells with insulin and IGF-I on the phosphorylation of tyrosine kinase receptors and downstream proteins.** (A,B) Total cell lysates from MDA-MB-435+mock cells and MDA-MB-435+mock stimulated (24h) with insulin or IGF-1 were obtained and analyzed by Western-blot for phospho-IR(Tyr1150-51)/phospho-IGF-IR(Tyr1135-36), IR, IGFR, Akt, phospho-Akt (Ser 473), ERK 1/2, phospho-ERK 1/2, β-catenin and E-cadherin. Increased phosphorylation levels of IR, IGF-IR, ERK 1/2 and Akt were observed after stimulation with insulin or IGF-I. Tubulin was used as a loading control.
(TIF)

Figure S3 **Effects of stimulation of Mock-transfected cells with insulin and IGF-I on cell invasion.** (A) Representative images of cell invasion through Matrigel using 8 mm pore of a polycarbonate membrane. Nuclei were stained with DAPI. No significant differences were observed on cellular invasion upon insulin and IGF-I stimulation of mock-transfected cells. (B) The bar graph shows the amount of cells/field. **Effects of stimulation with insulin and IGF-I on the fibronectin protein and mRNA expression levels, respectively**. (C) and (D) A slight increase of fibronectin protein expression levels were observed after stimulation with insulin or IGF-I, however, no significant changes were observed at the mRNA transcription levels after stimulation of MDA-MB-435+E-mock cells with insulin and IGF-I . **Effects of overexpression of MGAT5 on the IR expression levels of MKN45 cell line**. (E) Total cell lysates from MKN45+mock and MKN45+MGAT5 were obtained and analyzed by Western blot for IR. An increased expression of IR were observed after overexpression of MGAT5. Tubulin was used as a loading control.
(TIF)

Figure S4 **Subcellular localization of E-cadherin and β-catenin of Mock-transfected cells stimulated with insulin and IGF-I.** Cell monolayers from MDA-MB-435+mock stimulated (24h) with insulin or IGF-1 were fixed and stained for E-cadherin, β-catenin and nucleus (DAPI). No significant differences were observed on the β-catenin subcellular localization after insulin or IGF-I stimulation. The representative images were obtained by fluorescence microscopy. Bar = 10 µm.
(TIF)
