# Supplementary figures and images for: Insulin/IGF-I Signaling Pathways Enhances Tumor Cell Invasion through Bisecting GlcNAc N-glycans Modulation. An Interplay with E-Cadherin
Source: PLoS One. 2013 Nov 25;8(11):e81579. doi: 10.1371/journal.pone.0081579 (PMC3839884; doi:10.1371/journal.pone.0081579)

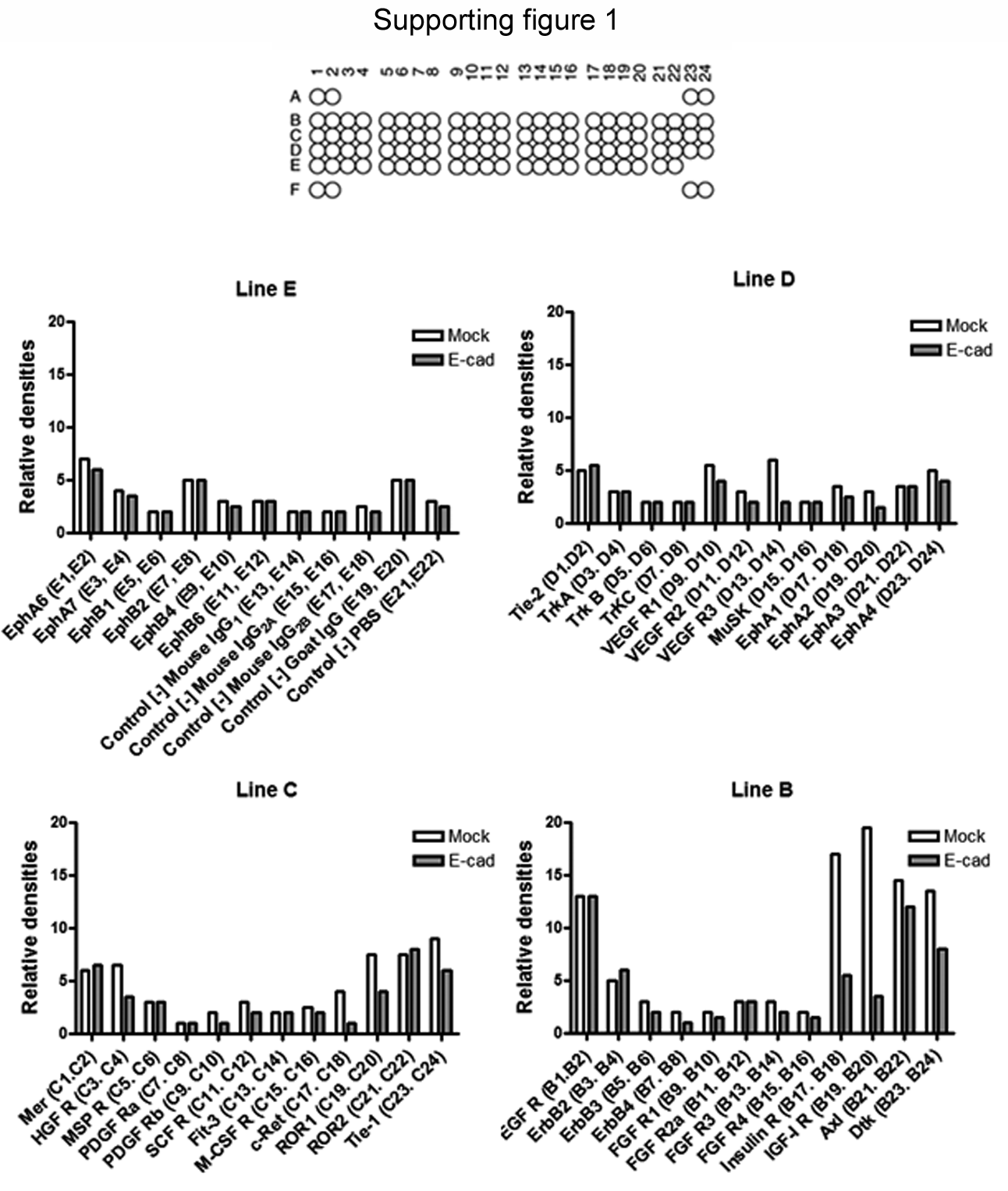

Supplement: Figure S1 — Effects of exogenous E-cadherin expression on the phosphoproteome profile. Total cell lysates from MDA-MB-435+mock and MDA-MB-435+E-cad were obtained and analyzed by Phospho-RTK array using 300 µg of proteins. The phosphor-RTK coordinates are shown on the top of figure illustrating the localization of the spots containing immobilized antibodies on the nitrocellulose membrane. The bar graphs show the relative densities of black dots. The most pronounced changes are observed in IR (coordinates B17 and B18) and IGF-IR (coordinates B19 and B20). (TIF) [file pone.0081579.s001.tif]

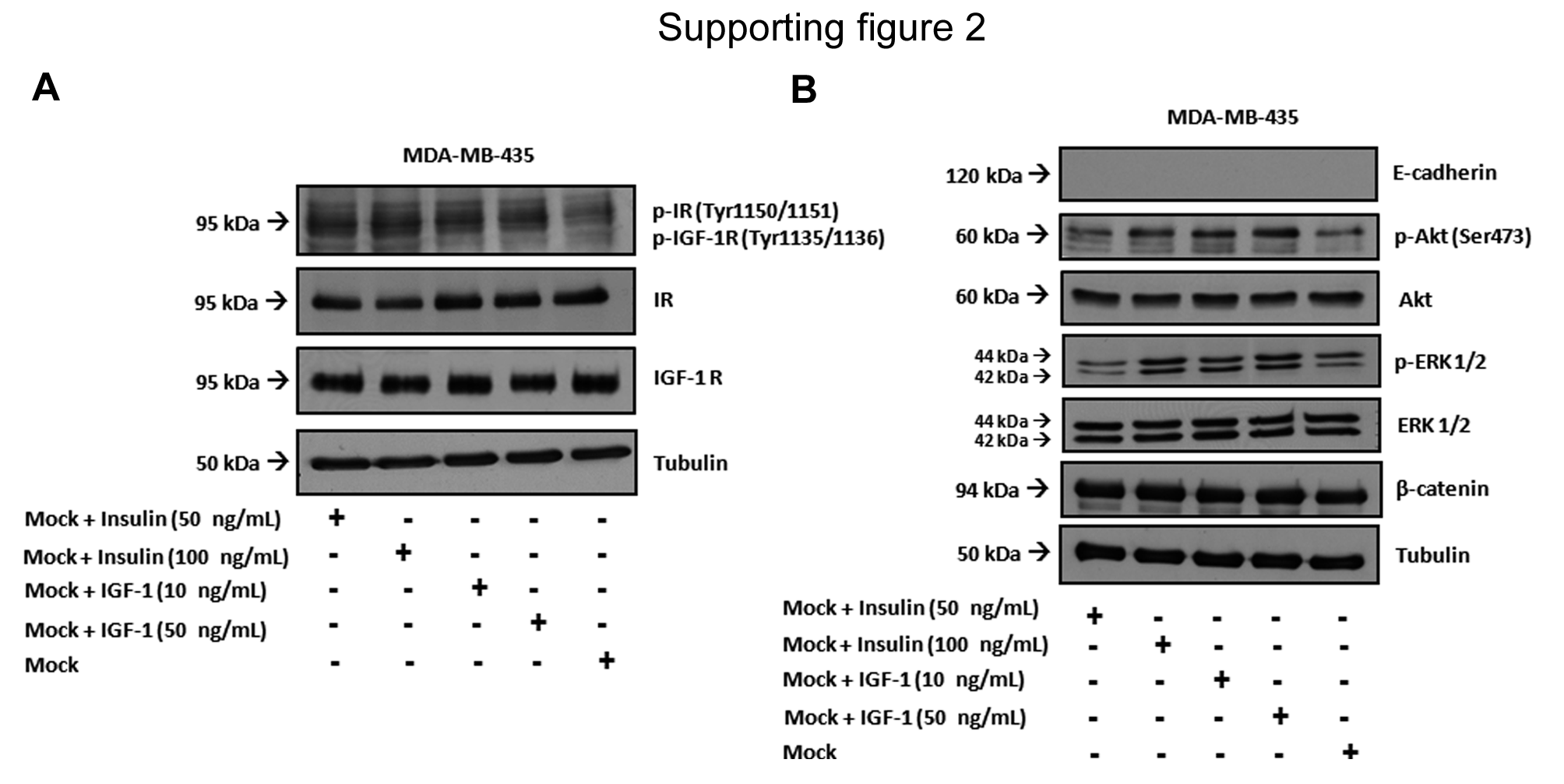

Supplement: Figure S2 — Effects of stimulation of Mock-transfected cells with insulin and IGF-I on the phosphorylation of tyrosine kinase receptors and downstream proteins. (A,B) Total cell lysates from MDA-MB-435+mock cells and MDA-MB-435+mock stimulated (24h) with insulin or IGF-1 were obtained and analyzed by Western-blot for phospho-IR(Tyr1150-51)/phospho-IGF-IR(Tyr1135-36), IR, IGFR, Akt, phospho-Akt (Ser 473), ERK 1/2, phospho-ERK 1/2, β-catenin and E-cadherin. Increased phosphorylation levels of IR, IGF-IR, ERK 1/2 and Akt were observed after stimulation with insulin or IGF-I. Tubulin was used as a loading control. (TIF) [file pone.0081579.s002.tif]

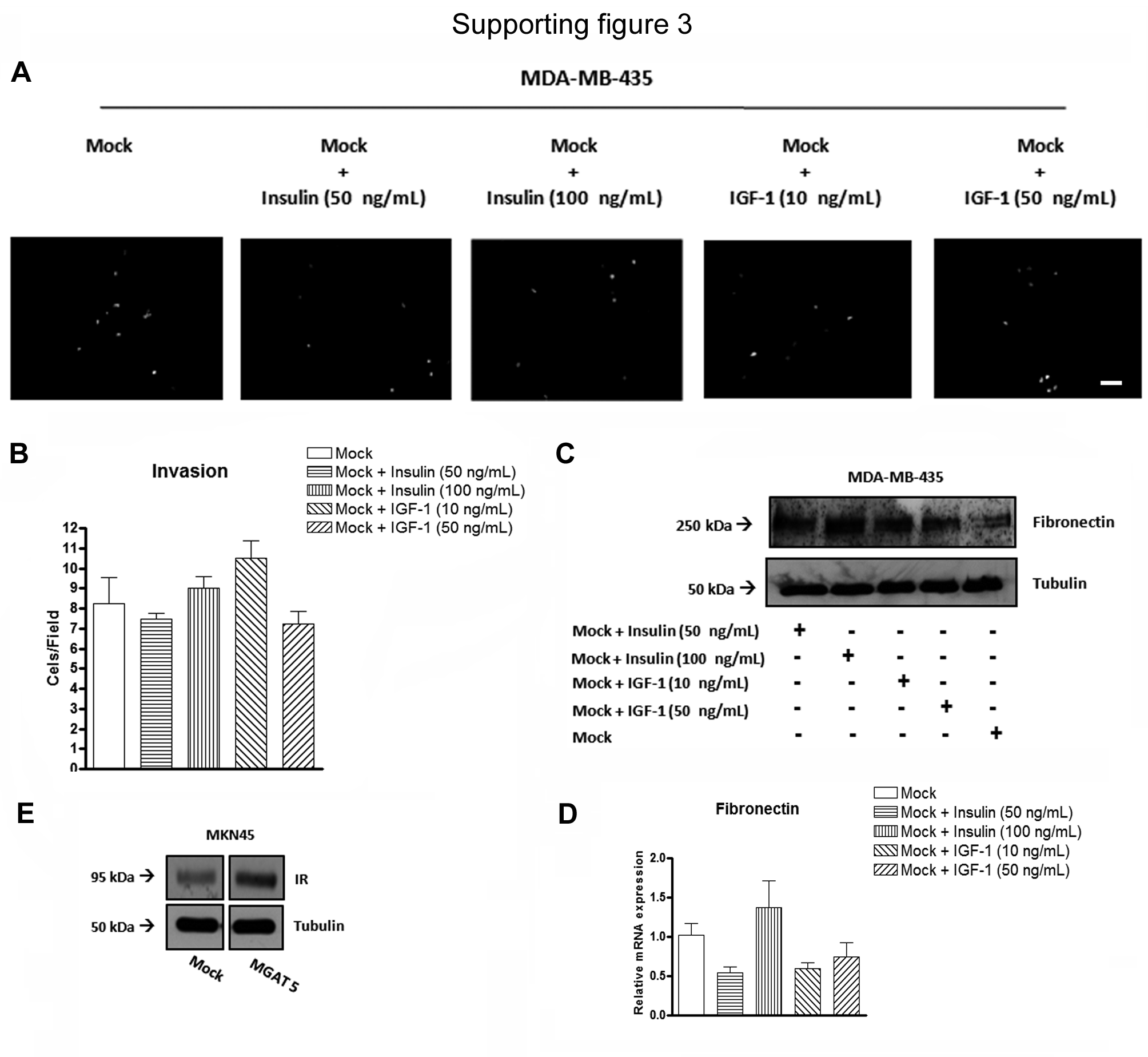

Supplement: Figure S3 — Effects of stimulation of Mock-transfected cells with insulin and IGF-I on cell invasion. (A) Representative images of cell invasion through Matrigel using 8 mm pore of a polycarbonate membrane. Nuclei were stained with DAPI. No significant differences were observed on cellular invasion upon insulin and IGF-I stimulation of mock-transfected cells. (B) The bar graph shows the amount of cells/field. Effects of stimulation with insulin and IGF-I on the fibronectin protein and mRNA expression levels, respectively. (C) and (D) A slight increase of fibronectin protein expression levels were observed after stimulation with insulin or IGF-I, however, no significant changes were observed at the mRNA transcription levels after stimulation of MDA-MB-435+E-mock cells with insulin and IGF-I . Effects of overexpression of MGAT5 on the IR expression levels of MKN45 cell line. (E) Total cell lysates from MKN45+mock and MKN45+MGAT5 were obtained and analyzed by Western blot for IR. An increased expression of IR were observed after overexpression of MGAT5. Tubulin was used as a loading control. (TIF) [file pone.0081579.s003.tif]

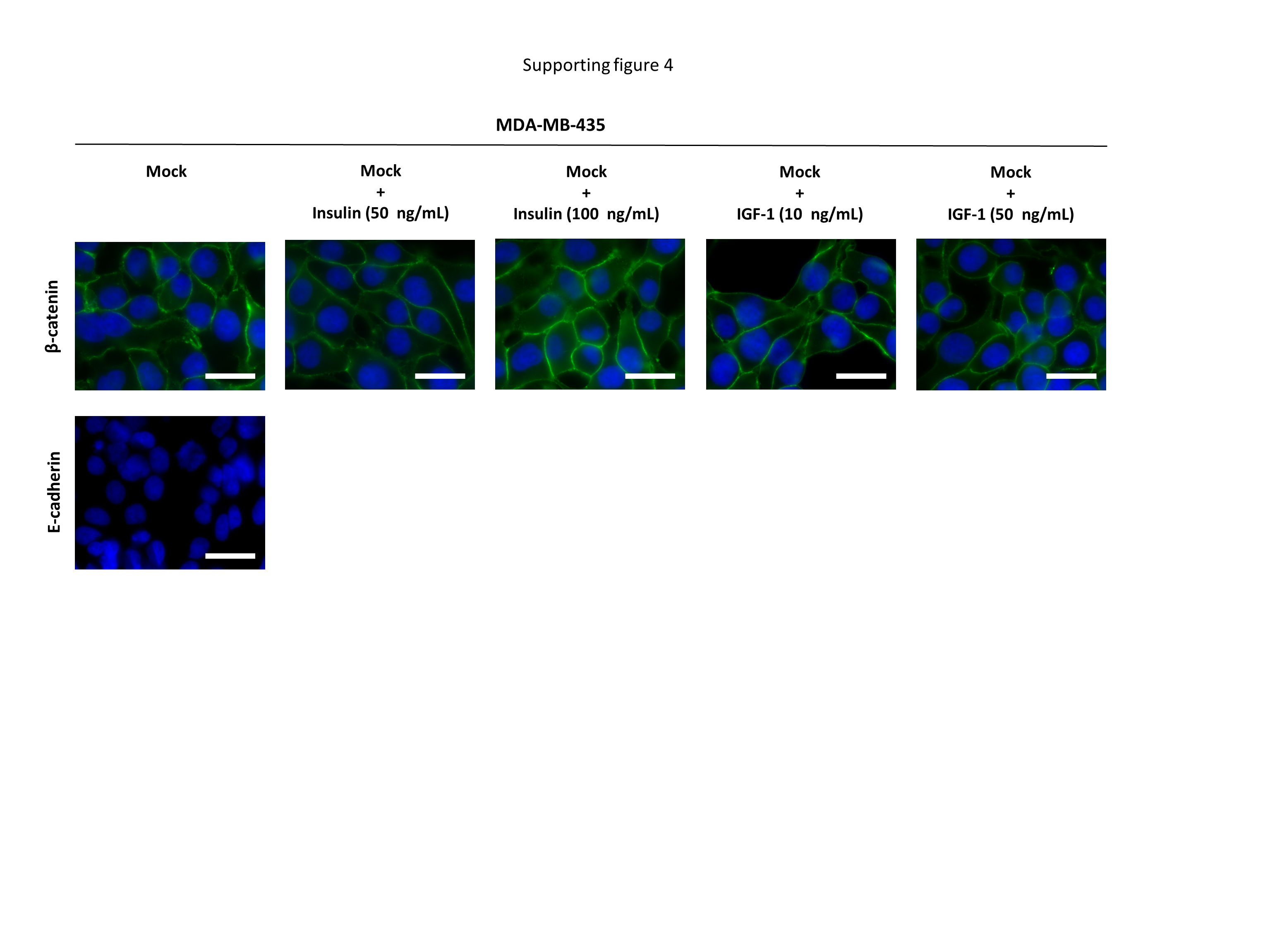

Supplement: Figure S4 — Subcellular localization of E-cadherin and β-catenin of Mock-transfected cells stimulated with insulin and IGF-I. Cell monolayers from MDA-MB-435+mock stimulated (24h) with insulin or IGF-1 were fixed and stained for E-cadherin, β-catenin and nucleus (DAPI). No significant differences were observed on the β-catenin subcellular localization after insulin or IGF-I stimulation. The representative images were obtained by fluorescence microscopy. Bar = 10 µm. (TIF) [file pone.0081579.s004.tif]
